# Fibres and cellular structures preserved in 75-million–year-old dinosaur specimens

**DOI:** 10.1038/ncomms8352

**Published:** 2015-06-09

**Authors:** Sergio Bertazzo, Susannah C. R. Maidment, Charalambos Kallepitis, Sarah Fearn, Molly M. Stevens, Hai-nan Xie

**Affiliations:** 1 Department of Materials, Imperial College London, South Kensington Campus, London SW7 2AZ, UK; 2 Department of Earth Science and Engineering, Imperial College London, South Kensington Campus, London SW7 2AZ, UK; 3 Department of Bioengineering, Imperial College London, South Kensington Campus, London SW7 2AZ, UK; 4 Institute for Biomedical Engineering, Imperial College London, South Kensington Campus, London SW7 2AZ, UK; 5 Present address: Department of Medical Physics and Biomedical Engineering, University College London, Malet Place Engineering Building, London WC1E 6BT, UK

## Abstract

Exceptionally preserved organic remains are known throughout the vertebrate fossil record, and recently, evidence has emerged that such soft tissue might contain original components. We examined samples from eight Cretaceous dinosaur bones using nano-analytical techniques; the bones are not exceptionally preserved and show no external indication of soft tissue. In one sample, we observe structures consistent with endogenous collagen fibre remains displaying ∼67 nm banding, indicating the possible preservation of the original quaternary structure. Using ToF-SIMS, we identify amino-acid fragments typical of collagen fibrils. Furthermore, we observe structures consistent with putative erythrocyte remains that exhibit mass spectra similar to emu whole blood. Using advanced material characterization approaches, we find that these putative biological structures can be well preserved over geological timescales, and their preservation is more common than previously thought. The preservation of protein over geological timescales offers the opportunity to investigate relationships, physiology and behaviour of long extinct animals.

## Abstract (web summary)

Soft tissue from vertebrate fossils has previously been documented, but only in exceptionally preserved specimens. Here, Bertazzo *et al*. describe structures consistent with collagen fibres and red blood cells from eight Cretaceous dinosaur bones, none of which are exceptionally preserved.

## Graphical abstract

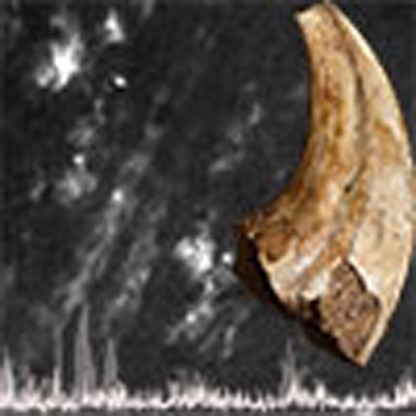

The preservation of vertebrate soft tissue has long been recognized and documented in exceptionally preserved fossils^1^,^2^,^3^,^4^,^5^,^6^,^7^,^8^,^9^,^10^,^11^,^12^. Recent research has suggested that original components of soft tissues such as skin^1^,^10^, feathers and other integumentary structures^2^,^3^,^4^,^5^,^6^,^7^,^8^,^9^, and muscle fibres^11^,^12^ may be preserved in these exceptional fossils. For example, still-soft, flexible material was recovered after demineralization of well-preserved bones from the Late Cretaceous dinosaur *Tyrannosaurus*^13^, whereas proteinaceous material was found to be preserved in another dinosaur, *Brachylophosaurus*^14^. Haemoglobin fragments were found in the abdomen of a beautifully preserved Eocene mosquito^15^, and degraded eumelanin was recovered in the integument of an Eocene turtle^16^.

Models proposed to account for such preservation indicate that it should be the exception rather than the rule^11^,^12^,^17^,^18^,^19^. In particular, it has long been accepted that protein molecules decay in relatively short periods of time and cannot be preserved for longer than 4 million years^19^,^20^. Therefore, even in cases where organic material is preserved, it is generally accepted that only parts of original proteins are preserved^15^,^16^ and that the full tertiary or quaternary structure has been lost.

Here, we examined eight dinosaur bones from the Cretaceous period, none of which are exceptionally preserved. We used electron microscopy and a focused ion beam (FIB), as part of a novel method to prepare samples for mass spectrometry. First, with a scanning electron microscope (SEM), we observed, in four different samples, structures resembling calcified collagen fibres from modern bone; in three other samples, structures enriched in carbon; and in two of our samples, structures that resemble erythrocytes from birds. Serial sectioning of one sample presenting fibres and of one presenting the erythrocyte-like structures revealed that these fibres are less dense than the matrix surrounding them and that an internal structure is present inside the erythrocyte-like structures. With a transmission electron microscope (TEM) we observed that the fibres show ∼67 nm banding, which could possibly be considered collagen fibre remains. Finally, using mass spectrometry, we found peaks that are consistent with fragments of amino acids present in collagen. The spectra obtained from the erythrocyte-like structures are surprisingly similar to the spectra obtained from the whole blood of an extant emu.

This synergistic approach to the application of the state-of-the-art materials analysis has therefore demonstrated utility in the study of fossils. A better understanding of the preservation of soft tissues and the discovery of these in ordinary, unexceptionally preserved fossils, could pave the way for biochemical and cellular investigations of the remains of extinct animals, shedding light on aspects of their physiology and behaviour that have been previously inaccessible to palaeontologists.

## Results and Discussion

### SEM analysis

**Figure 1:**
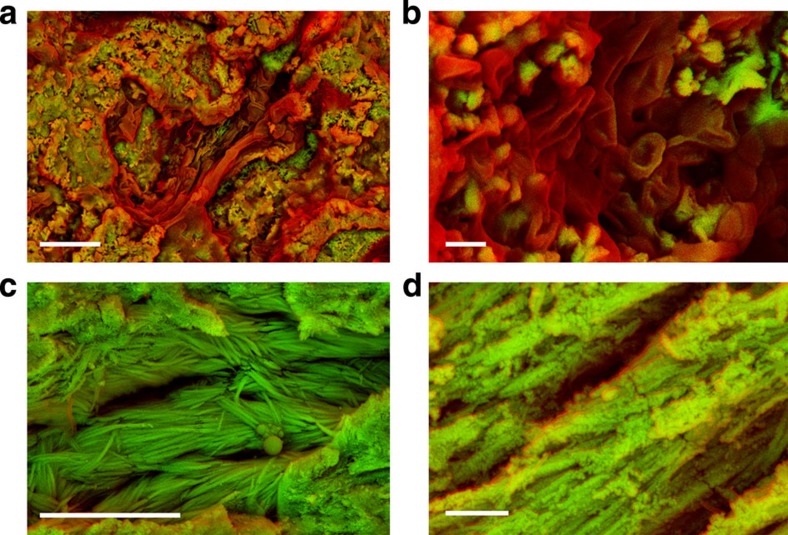
Density-dependent colour scanning electron micrographs of samples of NHMUK R12562, an ungual claw of an indeterminate theropod dinosaur, and NHMUK R4493, ribs from an indeterminate dinosaur. (**a**) Amorphous carbon-rich material (red) surrounded by dense material (green). Scale bar, 5 μm. (**b**) Erythrocyte-like structures composed of carbon surrounded by cement. Scale bar, 1 μm. For comparison, fixed blood from an emu (Dromaius) is shown in Supplementary Fig. 2c, d. Fibrous structures. Scale bar, 5 μm in (**c**) and 1 μm in (**d**).

We examined fossilized bones samples using SEM from eight dinosaur specimens (Supplementary Table 1) that show no external indication of soft tissue. Density-dependent colour SEM^21^ obtained from the surface of minute pieces broken off from samples with tweezers revealed numerous low-density structures suggestive of soft tissue in sample NHMUK (Natural History Museum, London, UK) R12562, an ungual claw of an indeterminate theropod dinosaur (Fig. 1 and Supplementary Figs 1,3,4). Among these structures ovoid structures highly reminiscent of avian erythrocytes^22^,^23^ (Fig. 1a,b) were imaged and ranged in length from 1.2 to 3.2 μm (average 1.8±0.4, *n*=35). In addition to the erythocyte-like structures, in four other specimens the SEM analysis also showed fibrous structures similar to calcified collagen fibres found in modern bone^24^,^25^,^26^ (Fig. 1c,d, Supplementary Table 1 and Supplementary Fig. 5).

Elemental analysis using energy dispersive X-ray spectroscopy (EDS) established that all these structures are enriched in carbon, in contrast with the surrounding denser tissue/cement, which is composed of carbon, oxygen, iron, silicon and aluminum (Supplementary Fig. 6). Martill and Unwin^27^ suggested that iron might be expected when looking for blood in fossils, and in a recent study, Greenwalt *et al*.^15^ found haemoglobin-derived porphyrin in the abdomen of a blood-engorged Eocene mosquito. Analysis of the material from the mosquito abdomen suggested it is 9% iron by weight. Given that red blood cells ordinarily contain only around 0.3% iron by weight, Greenwalt *et al*. suggested the unusual high percentage of iron measured may be due to a taphonomic concentration^15^. Our EDS analysis of the erythrocyte-like structures in sample NHMUK R12562 shows that iron is present in higher quantities in areas of the fossilized bone where no erythrocyte-like structures are preserved. This is to be expected because an iron concentration of only 0.3% by weight typical of a red blood cell is below the detection limit of EDS^28^. To confirm this hypothesis, we carried out EDS on emu blood and also did not detect iron, thus confirming the detection limit of the technique (Supplementary Fig. 7).

### Internal structures investigated by FIB

**Figure 2:**
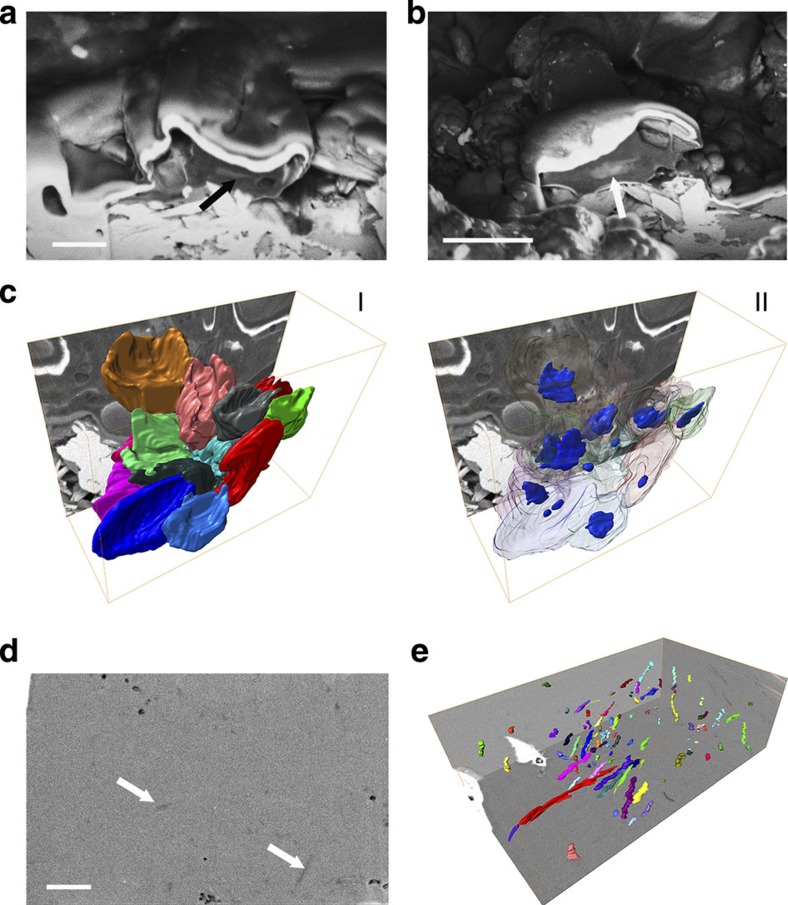
Scanning electron micrographs and 3D reconstructions from serial sections of erythrocyte-like structures and fibrous material. (**a**, **b**) SEM using a backscattering detector showing cross-sections of erythrocyte-like structures **c** in NHMUK R12562; arrows indicate dense internal material; scale bar, 0.5 μm. (**c**) 3D reconstruction of serial sections of an agglomeration of erythrocyte-like structures showing: I, upwardly concave external morphology and II, dense structures observed in the interior of the erythrocyte-like structures. (**d**) SEM using a backscattering detector from section of fossilized bone of NHMUK R4493; arrows indicate less dense zones inside bone matrix; scale bar, 1 μm. (**e**) 3D reconstruction of the less dense zones in **d**, showing elongated, fibre-like morphology and alignment. Random colours assigned to individual fibres to differentiate between them.

To evaluate the internal structure of the features imaged by SEM, we initially used a FIB to section the erythrocyte-like structures from NHMUK R12562 and fibrous structures present in NHMUK R4493, rib fragments from an indeterminate dinosaur (Supplementary Fig. 1). Interestingly, denser structures (as imaged by the backscattering detector) were observed inside the erythrocyte-like structures (Figs 2a,b). To further investigate these features, three-dimensional (3D) reconstruction of serial sections of an agglomeration of erythrocyte-like structures (Fig. 2c and Supplementary Movie 1) showed that each was concave, whereas the dense internal features resembled nuclei and could be clearly distinguished within each of them.

The serial sections from NHMUK R4493 showed less dense linear zones surrounded by a denser matrix within the samples (Fig. 2d). 3D reconstruction of the less dense zones (Fig. 2e and Supplementary Movie 2) clearly showed that these are 3D fibres with an average diameter of 54±9 nm (*n*=30) and are preferentially aligned in a specific direction (Supplementary Fig. 8).

### TEM analysis

**Figure 3:**
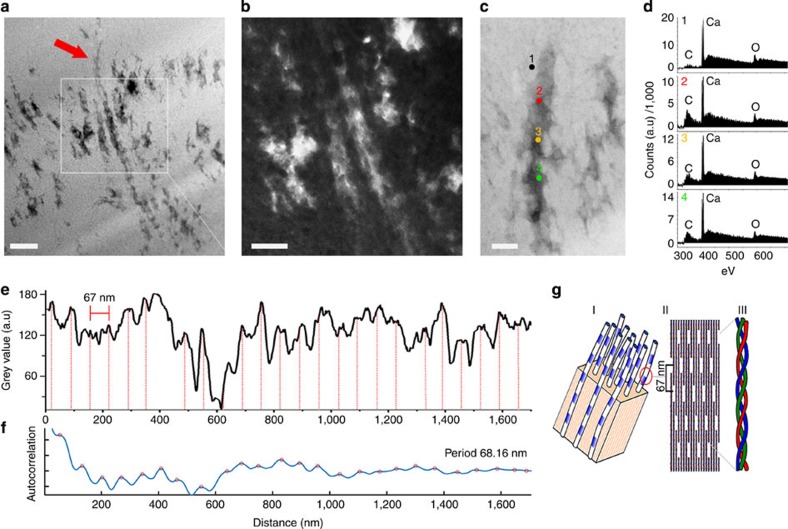
Scanning transmission electron microscopy (STEM) analysis of NHMUK R4493. (**a**) Bright-field STEM micrograph depicting fibre fragments showing a banded pattern consistent with banding typically observed in collagen fibrils. The arrow indicates the fibre analysed in **e** and **f**. Scale bar, 200 nm. (**b**) Dark-field STEM micrograph showing detail of fibres in **a**. Scale bar, 100 nm. (**c**) STEM of fibre analysed by electron energy loss spectroscopy (EELS) indicating spectra locations. Scale bar, 50 nm. (**d**) EELS spectra showing a carbon peak on the fibre. A smaller peak is seen on the adjacent material. (**e**) Grey intensity distribution over fibre (indicated by the arrow) in **a**. Vertical lines in red are spaced 67 nm apart and generally correspond with peaks in grey intensity. (**f**) Periodicity characterization of **e** confirming the ∼67 nm banding. (**g**) Diagram representing the structure of a generic collagen molecule that produces 67 nm banding; I banded collagen fibrils surrounded by bone mineral matrix; II individual fibrils are composed of numerous collagen molecules arranged to produce 67 nm banding; III the canonical collagen triple helix.

To evaluate the ultrastructure of these fibres, TEM was carried out on sections from NHMUK R4493, NHMUK R4243, an astragalus of a hadrosaurid, NHMUK R4249, an ungual phalanx of a hadrosaurid and NHMUK R4864, a hadrosaurid tibia (as a control, rabbit bone (Supplementary Fig. 9) was also prepared using the same method). All samples were prepared by FIB, a method that would preserve the mineral and organic material^21^. The sections were obtained from the interior of each sample, ruling out modern surface contamination. Unexpectedly, from three of the samples (NHMUK R4493, NHMUK R4249 and NHMUK R4243) TEM micrographs showed obvious fibrous structures (Fig. 3a,b,c and Supplementary Fig. 10) containing carbon (Fig. 3c,d). One sample (NHMUK R4493) also showed, for the first time in a dinosaur bone, a clear ∼67 nm banding, that is typical of the banding observed in collagen (Fig. 3e), for the length of the preserved fibre.

In modern day bone collagen, the ∼67 nm banding is considered a diagnostic ultrastructure characteristic and arises from the arrangement of fibrils to form the quaternary structure of the collagen protein^29^,^30^,^31^,^32^,^33^,^34^,^35^,^36^,^37^,^38^,^39^,^40^,^41^ (Fig. 3f). Although 67 nm banding can be considered, in some cases, a finger print of specific proteins, this method is not able to show the chemical composition of the structures imaged. If collagen is completely degraded, this banding is no longer seen, due to loss of the quaternary structure of the protein. Therefore, the observation of a ∼67-nm banding in the fibrous structures of fossilized samples here is very exciting, as it is consistent with a preservation of the ultrastructure of putative collagen fibres over a time period of 75 million years. Before this finding, the oldest undegraded collagen recorded (based on mass spectrometry sequencing and peptide fingerprinting) was about 4 million years old^20^.

### Mass spectrometry analysis

**Figure 4:**
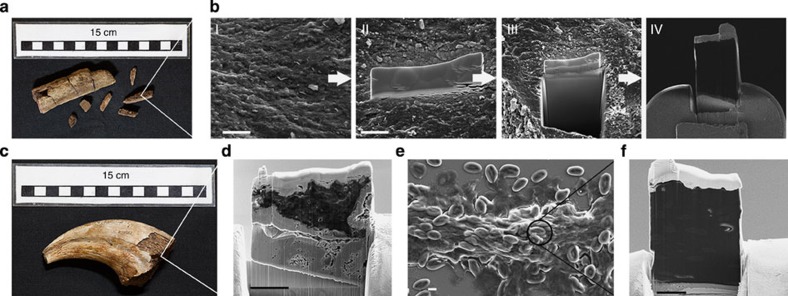
Sample preparation by focused ion beam (FIB) for mass spectroscopy analyses of NHMUK R4493, NHMUK R12562 and fixed emu blood. (**a**) NHMUK R4493 with sampled location. (**b**) SEM of FIB sample preparation sequence: I, sample surface; II, platinum protecting layer; III, trench milling; IV, sample on copper grid holder ready for mass spectra acquisition. (**c**) NHMUK R12562 with sampling location. Scale bar, 5 μm. (**d**) Sample on grid holder before mass spectra acquisition. Scale bar, 5 μm. (**e**) SEM image of fixed emu blood with sampling location. (**f**) Sample on grid holder before mass spectra acquisition. Scale bar, 5 μm. The mass spectrum was obtained from the fresh surface in IV (**d** and **f**).

Finally, a thick section (∼20 × 15 × 5 μm^3^) of samples was extracted using FIB (see Fig. 4 for details of sample preparation) and analysed with Time-of-Flight Secondary Ion Mass Spectrometry (ToF-SIMS) to probe for the presence of amino acids. Sections were obtained from an agglomeration of erythrocyte-like structures and cement surrounding these from specimen NHMUK R12562, fixed emu blood, three fossils showing calcified fibres (NHMUK R4493, NHMUK R4249, NHMUK R4864), rabbit bone and a fossil not presenting sign of calcified fibres (NHMUK R12562). As a control, a mass spectrum from the copper grid holding the samples was also obtained.

Extraction of a fresh surface for ToF-SIMS analysis by the FIB placed inside the electron microscope guarantees correct localization of sampling and provides a clean, smooth surface for analysis that eliminates topographical artefacts common to ToF-SIMS measurements^42^. Moreover, in the case of the cement from specimen NHMUK R12562 and NHMUK R4493 (where the bone was directly sampled), this method rules out the possibility of modern contamination, as the surface exposed is inaccessible to any contaminant.

**Figure 5:**
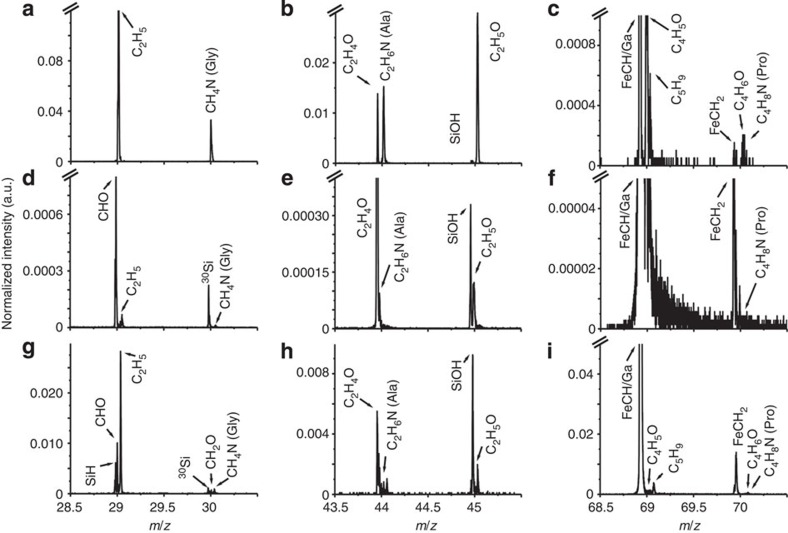
Mass spectra detail of NHMUK R4249 (sample with banded fibres), NHMUK R4493 (sample with 67 nm banded fibres) and NHMUK R12562 cement. (**a**, **d** and **g**) Peaks are associated with glycine fragments. (**b**, **e** and **h**) Peaks are related to alanine fragments. (**c**, **f** and **i**) Peaks are related to proline fragments.

The positive mass spectrum obtained from NHMUK R4493 showed peaks corresponding to fragments of the amino acids glycine, alanine, proline and others^14^,^43^,^44^ (Fig. 5 and Supplementary Fig. 11; for attribution of peaks see Supplementary Table 2). Moreover, several peaks present in the spectrum from rabbit bone and the fossil samples showing calcified fibres are not present in the results for the fossil without calcified fibres or on the negative control from the copper grid holding the samples (Supplementary Figs 12 and 13). Detection of fragments of the amino acids normally found in collagen supports the results obtained from TEM analysis where the ∼67 nm banding is consistent with potential preservation of the original quaternary structure of the protein.

**Figure 6:**
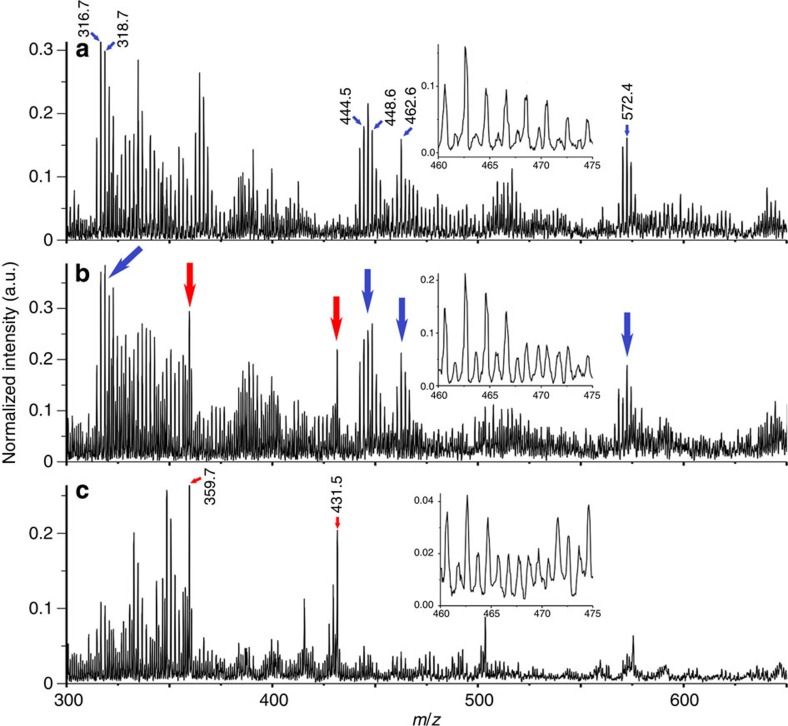
Mass spectra of fixed emu blood, erythrocyte-like structures present in NHMUK R12562 and cement surrounding these erythrocyte-like structures, all prepared by FIB. (**a**) Mass spectrum of emu blood with inset detailed region between 460 and 475 *m*/*z*. (**b**) Mass spectrum of erythrocyte-like structures present in NHMUK R12562, with inset detailed region between 460 and 475 *m*/*z*. (**c**) Mass spectrum of cement surrounding the erythrocyte-like structures, with inset detailed region between 460 and 475 *m*/*z*. Blue arrows indicate the regions of main peaks in each spectrum that are present only in the mass spectra of fixed emu blood and erythrocyte-like structures. Red arrows indicate the regions of main peaks in each spectrum that are present only in the mass spectra of erythrocyte-like structures and cement surrounding erythrocyte-like structures.

The positive mass spectra obtained from the erythrocyte-like structures of NHMUK R12562 were compared with spectra obtained from emu blood fixed in paraformaldehyde and with the cement surrounding the structures (Fig. 6). The spectra obtained from four different regions of the dinosaur bone containing erythrocyte-like structures are surprisingly similar to the spectra obtained from emu blood.

As expected, spectra obtained from emu blood (Fig. 6a) indicate the presence of several components, as the spectra were obtained from sections that comprised whole blood. Some of the main peaks could be tentatively assigned to components present in whole blood such as: 318 *m*/*z* to folic acid^45^; 462 *m*/*z* to hydroxycholesterol^46^,^47^ and the peak at 572 *m*/*z* to ceramide (usually present in cell membranes^48^). The same pattern of peaks can be seen from the spectra obtained from the erythrocyte-like structures (Fig. 6b) and this is clearer when a higher magnification is applied to those spectra (Fig. 6a,b inset). Moreover, it is clear that the mass spectrum from the cement surrounding erythrocyte-like structures (Fig. 6c) is different from both the spectrum from erythrocyte-like structures and that from emu blood. It is particularly interesting that the mass spectrum from the erythrocyte-like structures has some features of both the mass spectra from emu blood and cement (Fig. 6b).

The presence of several protein residues preserved in the erythrocyte-like structures of NHMUK R12562 could explain differences between the spectra obtained by us and previous spectra obtained from fossilized blood samples such as that obtained by Greenwalt *et al*.^15^, where only haemoglobin-derived porphyrin was preserved in the fossil record. Finally, it is clear that the spectrum from cement is different from the spectra obtained from emu blood and from erythrocyte-like structures. Similarities between cement and the erythrocyte-like structures are expected because the cement is also present in the sample of the erythrocyte-like structures and fragments of the putative proteins are likely present in the cement, even if they cannot be visualized by electron microscopy. The mass spectra obtained from emu whole blood are slightly different in comparison with those of the erythrocyte-like structures, which is to be expected because of the presence of organic components derived from additional blood proteins that have not degraded in the emu sample, but are absent in the erythrocyte-like structures, and also because the emu blood was fixed with paraformaldehyde.

Partial least square—discriminant analysis (PLS-DA) of mass spectra from four dinosaur regions presenting the erythrocyte-like structures samples and four emu samples, collected from different regions and from a cement sample was carried out. The cross-validated PLS-DA were carried out in the region between 300 and 700 *m*/*z* (stressing, any organic differences between the components of the samples (Supplementary Figs 14 and 15)). The spectra from emu blood samples and those from areas of NHMUK R12562 containing erythrocyte-like structures are enclosed within the 95% confidence ellipse and, as a group, may be compared with the cement sample, which falls outside the ellipse. This is another indication that the biochemical fingerprints of the spectra of emu blood samples and of areas of NHMUK R12562 containing erythrocyte-like structures are very much alike.

Within the dinosaur samples on average, the erythrocyte-like structures are ∼2 μm in length. This is somewhat smaller than erythrocytes of birds, which range from 9 to 15 μm in length^22^,^49^; emu blood cells in our sample were 9±2 μm (*n*=17). The structures consistent with putative erythrocytes in the fossil could well have been deformed and it is quite probable that these structures have undergone some shrinkage during fossilization.

Red blood cell size is known to correlate with metabolic rate in many vertebrate clades, including reptiles and birds^49^. Discoveries of red blood cells in a range of dinosaur taxa offer a potential independent method of assessing relative metabolic rates and may help to ascertain when and how the transition from ectothermy to endothermy took place on the avian stem lineage.

The potential for future research into the metabolic rate of extinct animals based on erythrocytes is promising because in this study, putative soft tissue (either erythrocyte-like structures, collagen-like, fibrous structures or amorphous carbon-rich structures (Supplementary Fig. 7)) was observed in six of our eight dinosaur specimens (Supplementary Table 1). Incredibly, none of the samples showed external indicators of exceptional preservation and this strongly suggests that the preservation of soft tissues and even proteins is a more common phenomenon than previously accepted.

These results show that to determine the presence of soft tissue in fossils a new synergistic approach needs to be applied where micro/nano-analytical methods are utilized to their full potential.

The common preservation of soft tissues could pave the way for cellular investigations of extinct animals, shedding light on aspects of physiology and behaviour that have been previously inaccessible to palaeontologists and inaugurating a new and exciting way to do paleontology.

## Methods

### Sample collection and preparation

Eight dinosaur specimens were selected for sampling (Supplementary Table 1). Specimens from the Dinosaur Park Formation and Lance Formation were selected because pore spaces in the trabecular bone tissue were not infilled with matrix or cement, making the removal of minute (∼0.5 × 0.5 mm^2^) samples of bone easier. Specimens representing both major dinosaurian clades (Ornithischia and Saurischia) and different osteological elements were chosen. Finally, specimens with broken edges were chosen to avoid contamination from glues and consolidants that may have been applied to the bone surface. Samples were taken using tweezers, with which a small piece of the original sample was broken off, providing a newly exposed surface for analysis.

### SEM analyses and FIB preparation

Samples were secured to an aluminium sample holder with carbon tape and carbon paste, which was then coated with 5 nm carbon (Quorum Technologies Turbo-Pumped Thermal Evaporators model K975X) and 5 nm chromium in a sputter coater (Quorum Technologies Sputter Coater model K575X).

### SEM and EDS analyses

Following the coating procedure, samples were imaged by SEM (Gemini 1525 FEGSEM) operating at 10 kV. The instrument was equipped with both an inlens detector, which recorded secondary electrons, and a backscatter electron detector. Density-dependent colour SEM images were obtained by imaging a region in inlens mode and subsequently imaging the same region in backscatter mode. Using ImageJ software, both images were stacked and the inlens image was assigned to the green channel, whereas the backscatter image was assigned to the red channel (for more details, see Supplementary Fig. 3). EDS were obtained in the regions of interest using the point and shoot mode, which focuses the beam on the selected region and provides a spatial resolution of 1 μm.

### FIB and SEM analyses

Following SEM analysis, samples were transferred to a SEM/Focused Ion Beam (Carl Zeiss—Auriga) with a gallium ion beam operated at 30 kV. Selected regions were milled using 4 nA current. Subsequently, the region exposed to milling was polished with 50 pA current and imaged by a backscattering detector with the electron beam operating at 1.5 V. The serial sections were obtained by sectioning samples at intervals of 20 nm (erythrocyte-like structures) or 10 nm (fossilized bone) and reconstructed using the Amira `software segmentation editor function.

### FIB and TEM sample preparation

Samples were transferred to a FIB (FEI FIB200-SIMS or a SEM/Focused Ion Beam Helios NanoLa 50 series Dual Beam). A region of 20 × 2 μm^2^ was coated with a 2-μm-thick platinum layer (93 pA at 30 kV) to protect the sample surface from the gallium beam during the milling and polishing processes. Two trenches of 20 × 10 × 10 μm^3^ (length × height × depth) were made using currents between 2.8 and 21 nA parallel to the platinum protective layer, creating a section with dimensions of approximately 20 × 15 × 5 μm^3^. The section was then thinned with the beam until it was 1 μm thick, using beam currents between 93 pA and 2.8 nA. After the rough thinning, the base of the section was cut using a current of 2.8 nA. With a micromanipulator needle, the resulting section was then attached to a FIB lift-out grid using platinum. The section was then thinned again, until it was ∼100-nm thick using beam currents between 28 pA and 2.8 nA. Finally, the resulting surface was polished with a gallium beam operated at 2 kV.

### Time-of-flight secondary ion mass spectrometry

After sectioning, the FIB grid and the sample were introduced into a FEI FIB200-SIMS and IONTOF TOF.SIMS^5^-Qtac^100^ LEIS. The mass spectrum was obtained on the face (∼15 × 20 μm^2^ area) of the section presented on Fig. 4a panel IV, d and f. High-resolution mass spectra were obtained from the samples using the analytical Bi_3_^+^ ion beam. Positive secondary ions were collected at ∼0.30 pA and 128 × 128 pixels were collected in each of the scanned areas for 64 s. Internal calibration of the mass spectra was carried out using the H^+^, C^+^, CH^+^, CH_2_^+^ and C_3_H_2_^+^ ion peaks.

### EDS analyses and electron energy loss spectroscopy (EELS)

Samples extracted and prepared by FIB were imaged and analysed at 200 kV on a JEOL 2100FX TEM (JEOL). EDS and EELS elemental analyses were obtained in TEM operating at 200 kV.

### Partial least squares—discriminant analysis

PLS-DA is an inverse least squares multivariate discrimination method, which is used for classification. It encompasses the PLS regression properties to fine-tune a principal component analysis model in order to highlight differences between multivariate data sets of different classes.^50^ Mass spectra of four samples with erythrocyte-like structures from dinosaur and four emu blood samples were imported to the PLS tool box (Eigenvector Research, Inc.) in Matlab for PLS-DA classification. The spectra were normalized and mean centred before being calculated through the model.^51^ As independent validation was not possible, leave-one-measurement-out cross-validation was used to validate and optimize the PLS-DA model complexity.

## Additional information

**How to cite this article:** Bertazzo, S. *et al*. Fibres and cellular structures preserved in 75-million-year-old dinosaur specimens. *Nat. Commun.* 6:7352 doi: 10.1038/ncomms8352 (2015).

## Supplementary Material

Supplementary Figures and Tables Supplementary Figures 1-15 and Supplementary Tables 1-2

Supplementary Movie 1 3D reconstruction of serial sections of an agglomeration of erythrocyte-like structures found on sample NHMUK R12562.

Supplementary Movie 2 3D reconstruction of serial sections of the less dense zones from NHMUK R4493 sample, showing elongated, fibre-like morphology and alignment.
