# Supplementary material for: Fibres and cellular structures preserved in 75-million–year-old dinosaur specimens
Source: Nat Commun. 2015 Jun 9;6:7352. doi: 10.1038/ncomms8352 (PMC4468865; doi:10.1038/ncomms8352)
Supplement: Supplementary Figures and Tables — Supplementary Figures 1-15 and Supplementary Tables 1-2 [file ncomms8352-s1.pdf]

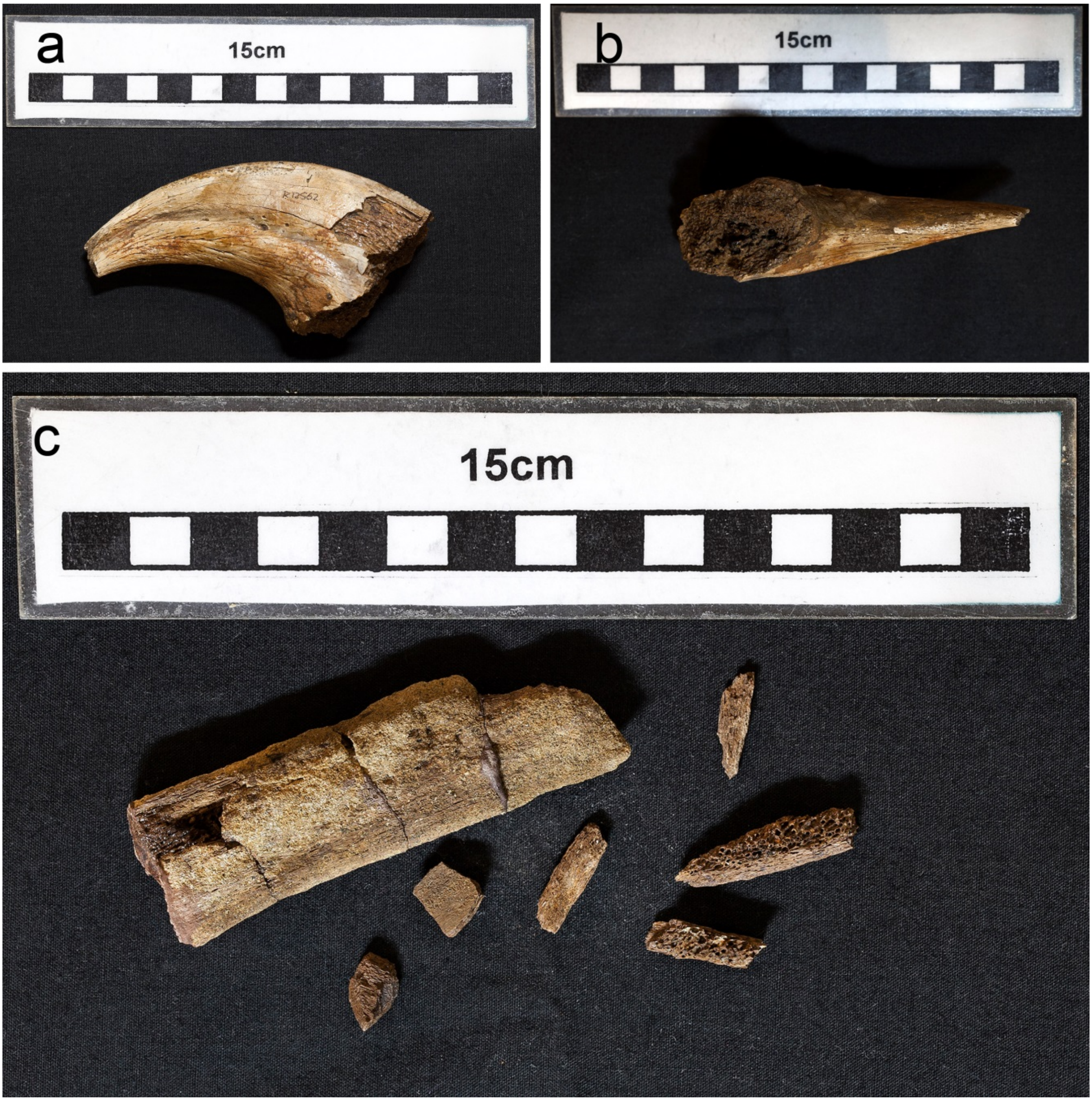

Supplementary Figure 1. **a**, **b**, NHMUK R12562, ungual claw of indeterminate theropod in **a**, lateral and **b**, plantar view. **c**, NHMUK R4493, rib fragments of an indeterminate dinosaur. Scale bars equal to 15 cm.

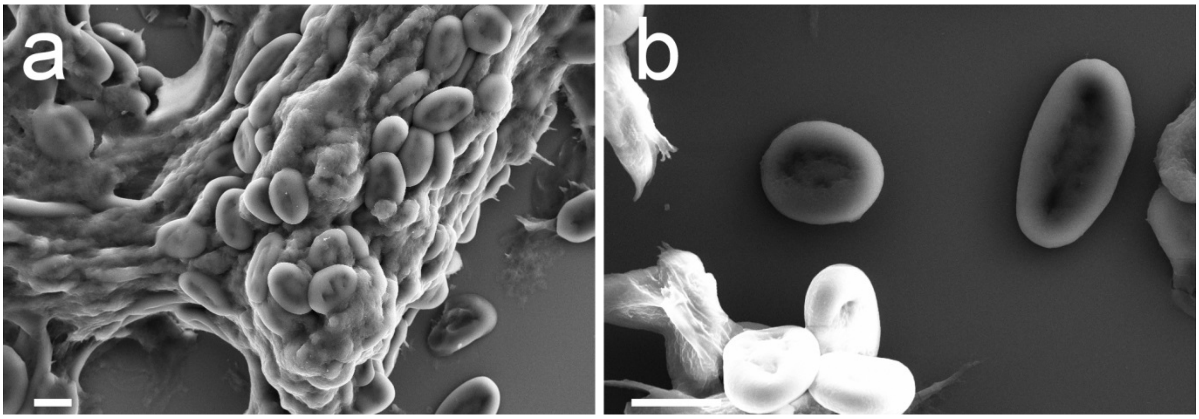

Supplementary Figure 2. Fixed blood from an emu (*Dromaius*), showing **a**, a clot with red blood cells and **b**, at higher magnification displaying the detail of red blood cells (Scale bar = 5 μm).

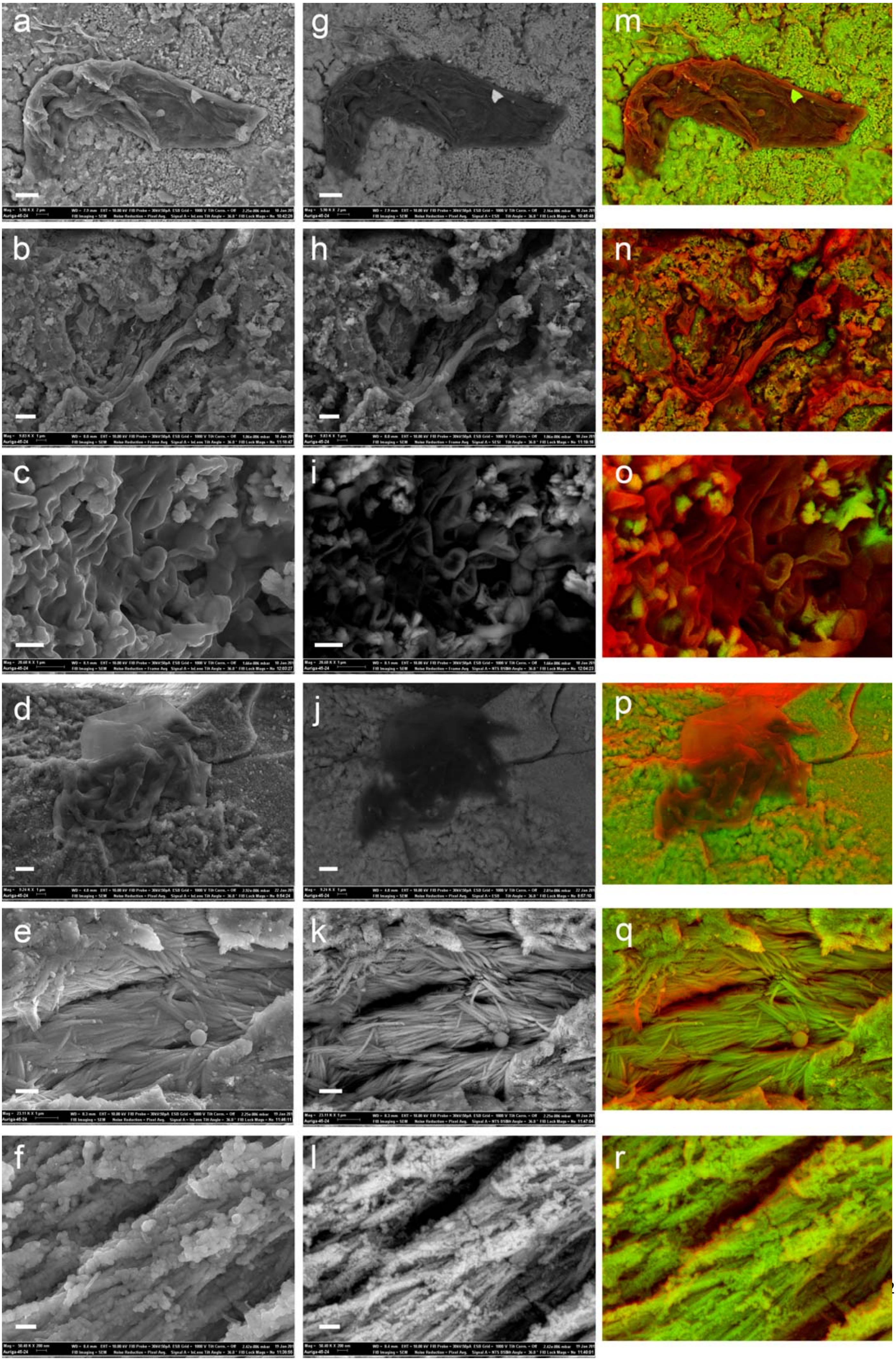
a
g
m
b
h
n
c
i
o
d
j
p
e
k
q
f
l
r

Supplementary Figure 3. Left-hand and centre column images were utilized to create each of the density-dependent colour scanning electron micrographs (DDC-SEM) in the right-hand column, which differ from standard "false color" SEM images. Using the "stack" command in ImageJ software, SEM micrographs acquired in secondary electron mode (**a**, **b**, **c**, **d**, **e** and **f**) were merged with SEM micrographs obtained from the identical region, but acquired in backscatter mode (**g**, **h**, **i**, **j** and **l**). The combined images were then converted into a single RGB image using ImageJ command "stack to RGB" with the backscatter image assigned to the green channel and the secondary electron mode image stacked on the red channel. This technique allowed for simultaneous visualization of both topography and density in a single image. The final output, images **m**, **n**, **o**, **p**, **q** and **r,** show denser material in green and the less dense material in orange/red. **a** and **g**, scale bar = 4 µm. **b**, **h**, **d**, and **j**, scale bar = 2 µm. **c**, **i**, **e** and **k**, scale bar = 1 µm. **f** and **l**, scale bar = 0.4 µm.

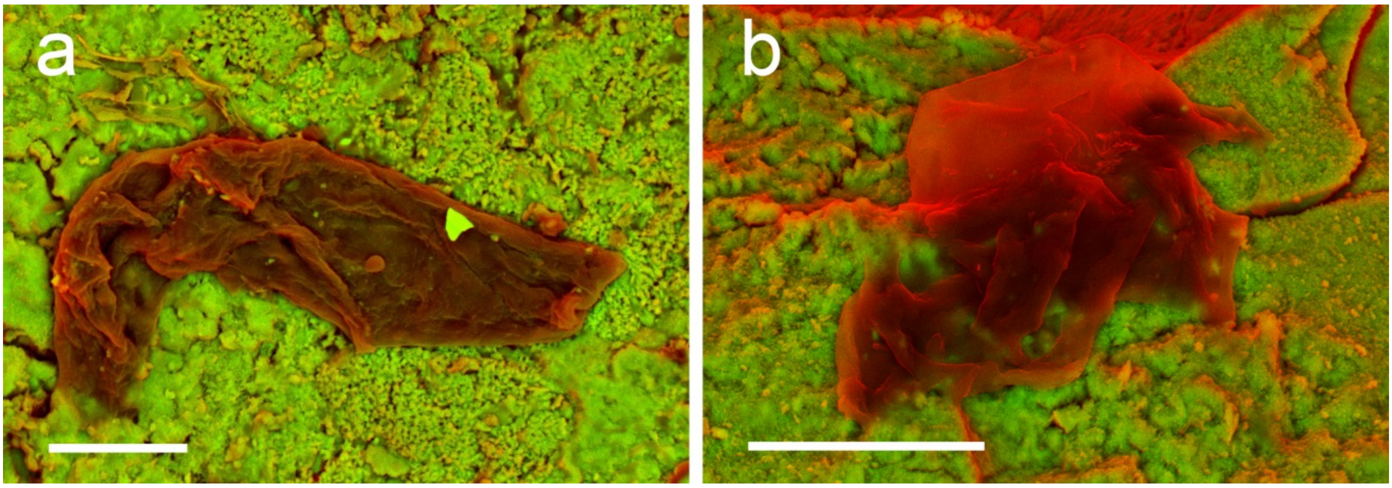

Supplementary Figure 4. a, Sample of NHMUK R12562, an ungual claw from indeterminate theropod, structure composed of carbon in red, over denser material (green). Scale bar = 10 µm. **b**, Sample of NHMUK R4493, structure rich in carbon in red. Scale bar = 10 µm.

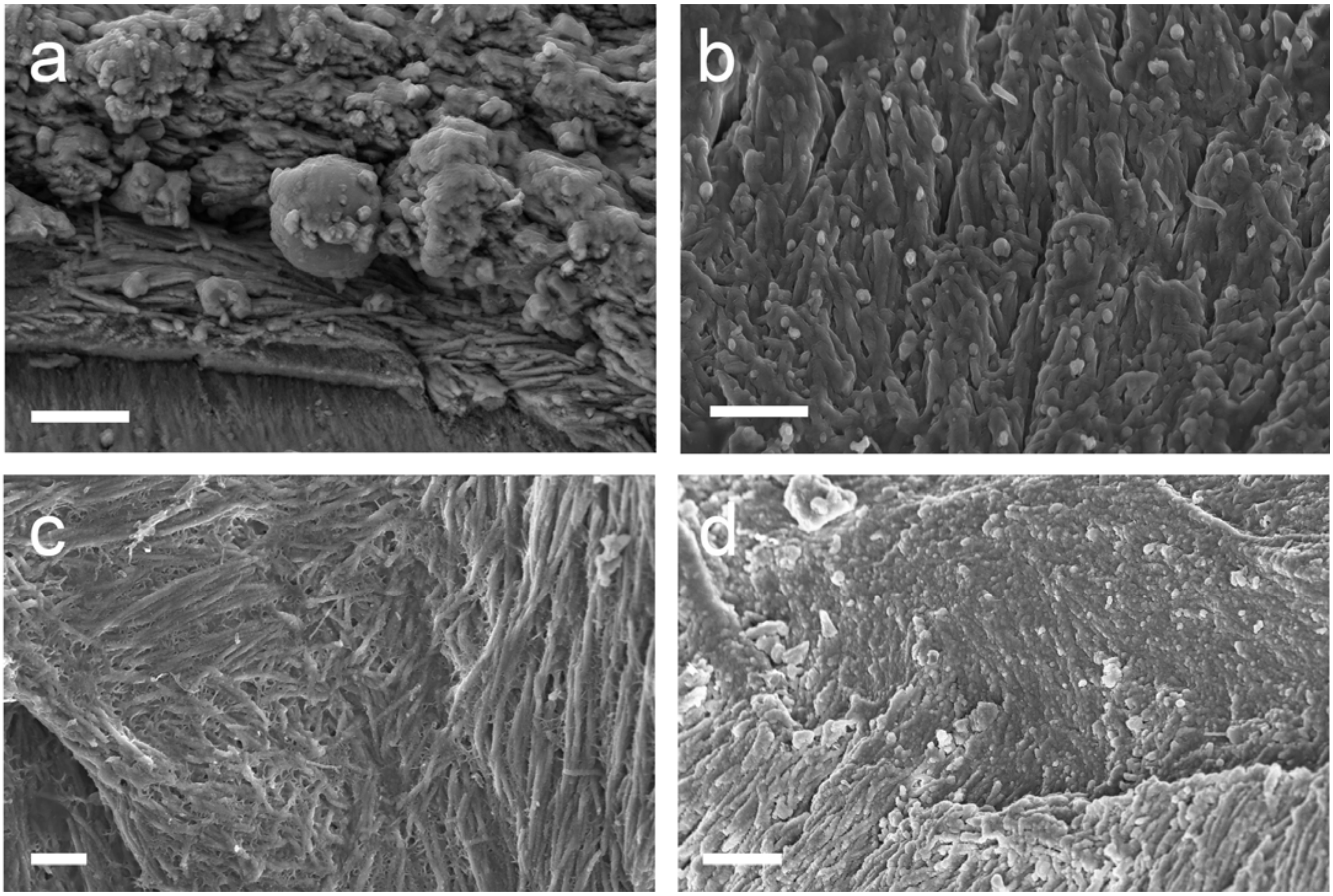

Supplementary Figure 5. Scanning electron micrographs of structures observed in dinosaur fossil samples: **a,** NHMUK R4249, ungual phalanx from a hadrosaurid. **b**, NHMUK R4243, astragalus from a hadrosaurid. **c**, **d**, sample NHMUK R4864, a tibia from a hadrosaurid. Scale bar = 2 µm.

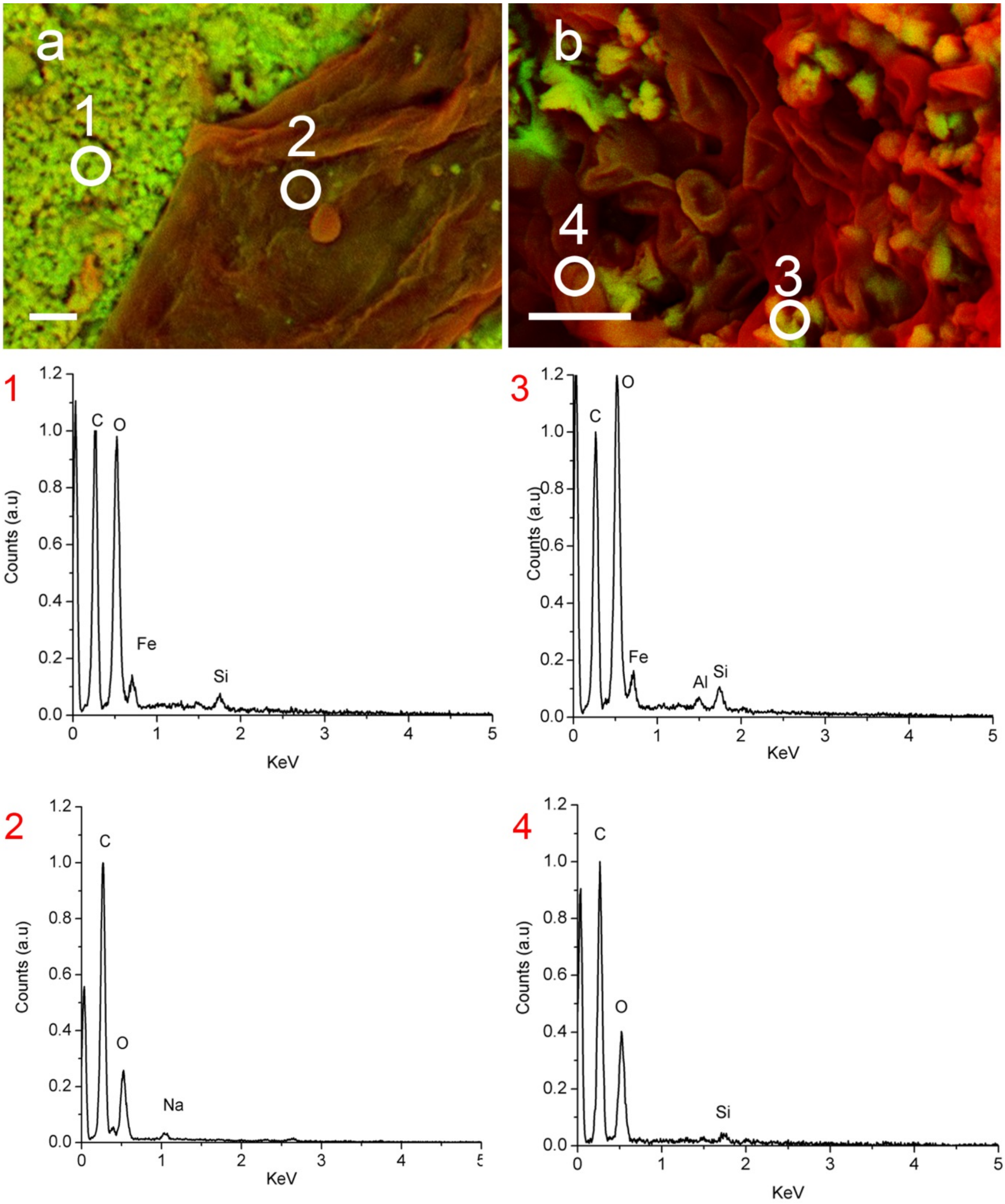

Supplementary Figure 6. Elemental analysis of structures found in specimen NHMUK R12562, an ungual claw from indeterminate theropod. **a**, **b**, DDC-SEM with regions (1 to 4 marked by circles) subsampled by energy-dispersive X-ray spectroscopy (EDS). Green corresponds to the denser material and red corresponds to the less dense material. Scale bar = 2 μm. Spectra were normalized to the peak value attributed to carbon.

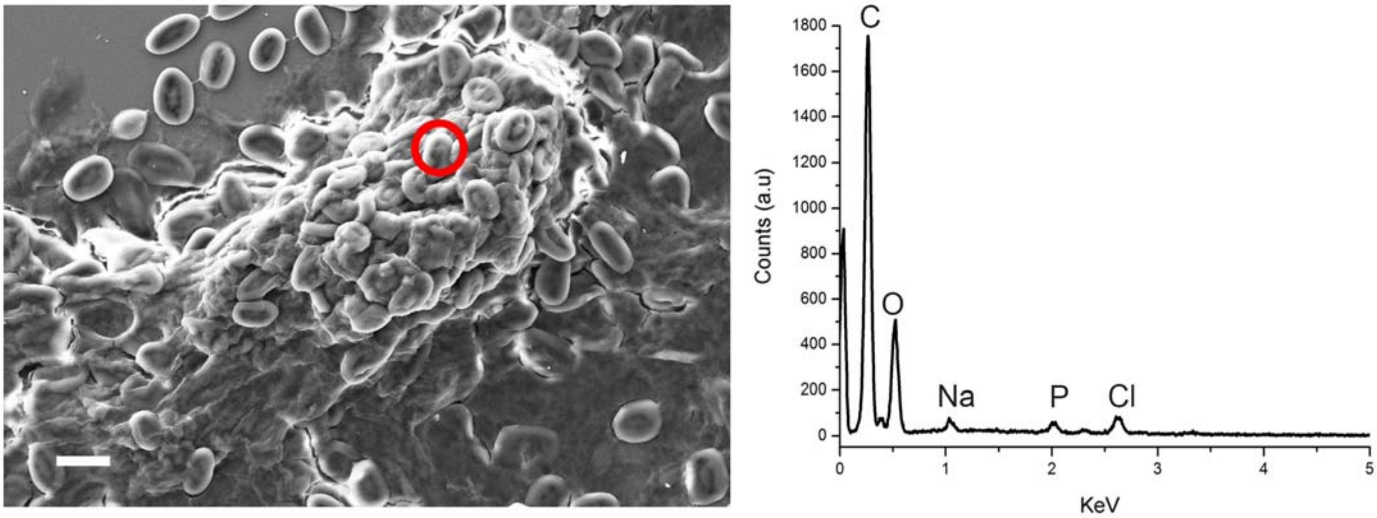

Supplementary Figure 7. Elemental analysis of fixed emu whole blood with the region in the circle subsampled by energy-dispersive X-ray spectroscopy (EDS). Scale bar = 10 μm.

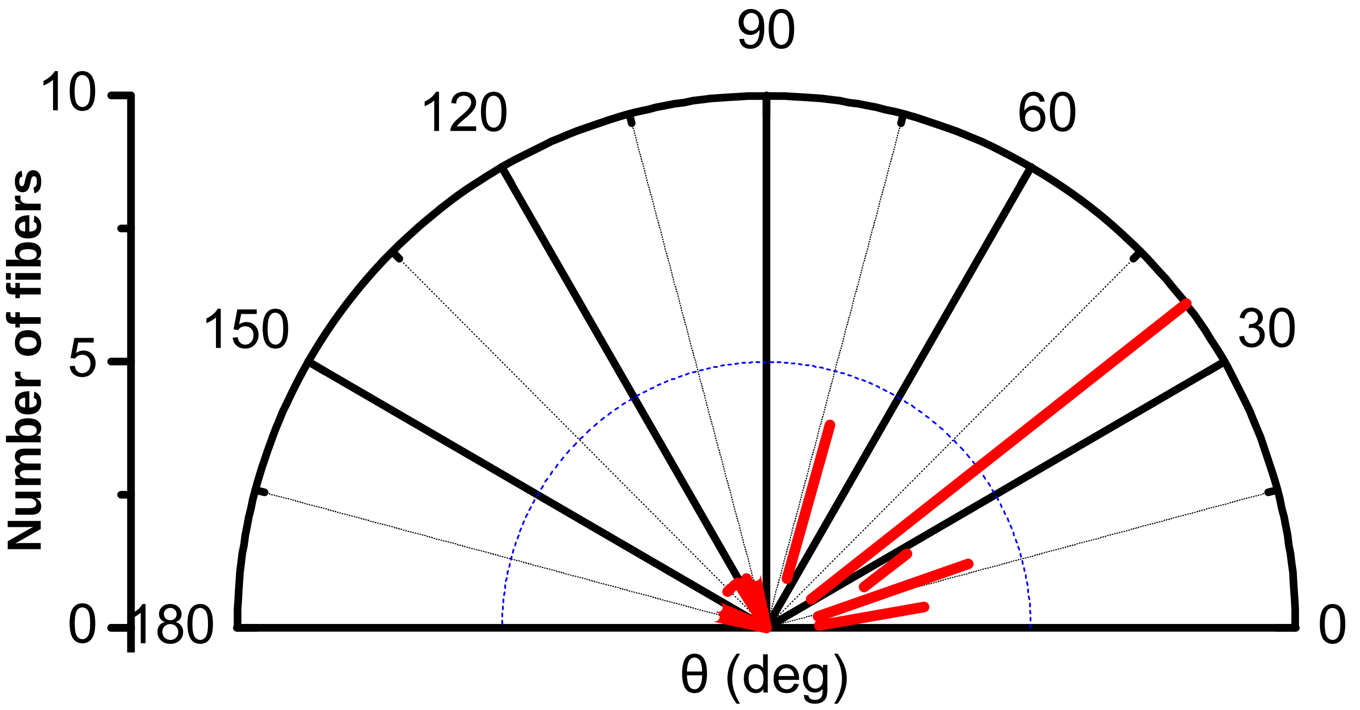

Supplementary Figure 8. Polar graph depicting the number of fibers of a specific angle related to the base of the image presented in Fig. 2E (n = 63).

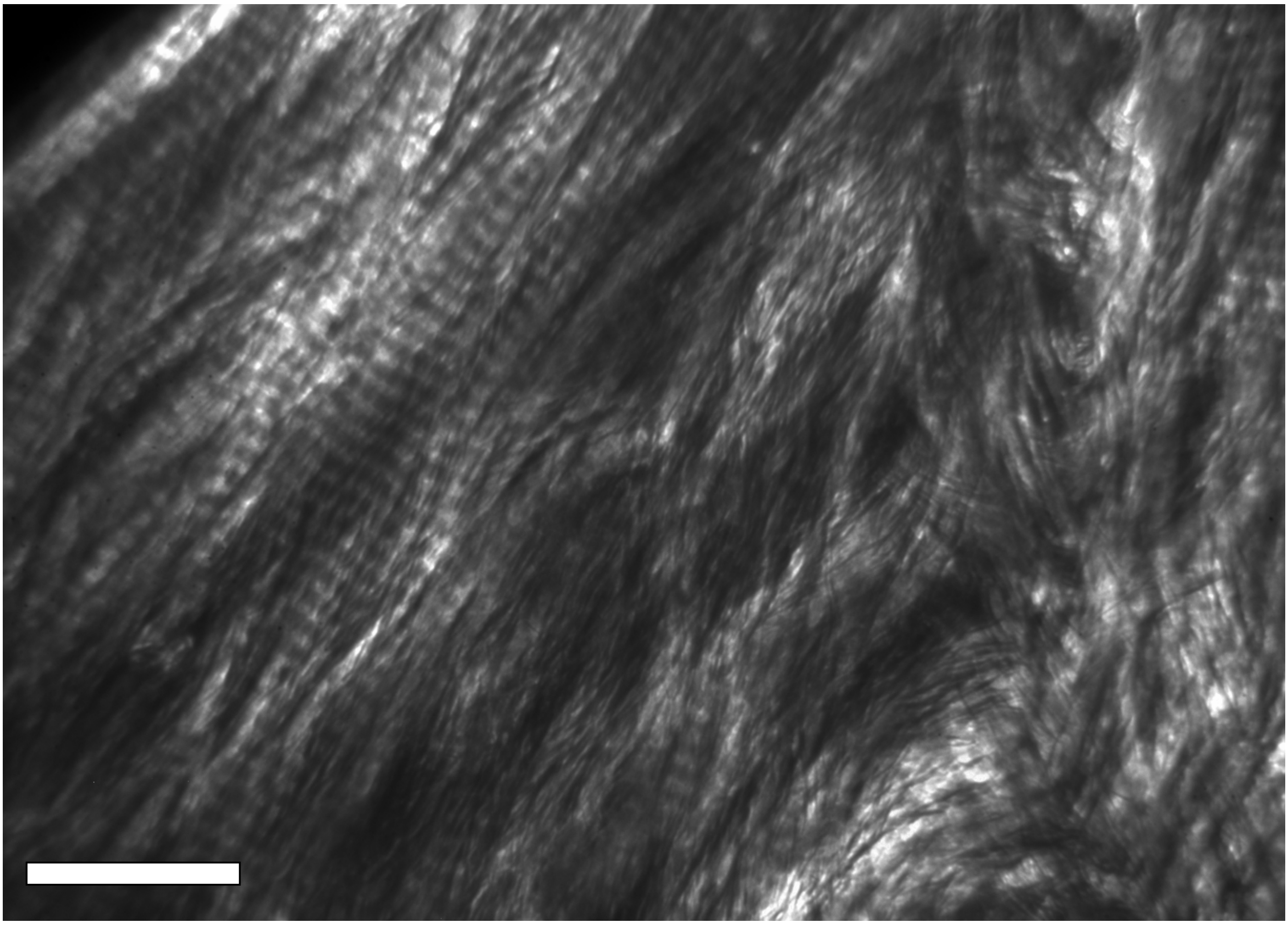

Supplementary Figure 9. TEM micrographs from rabbit bone depicting banded collagen fibers and adjacent mineral. Scale bar = 500 nm.

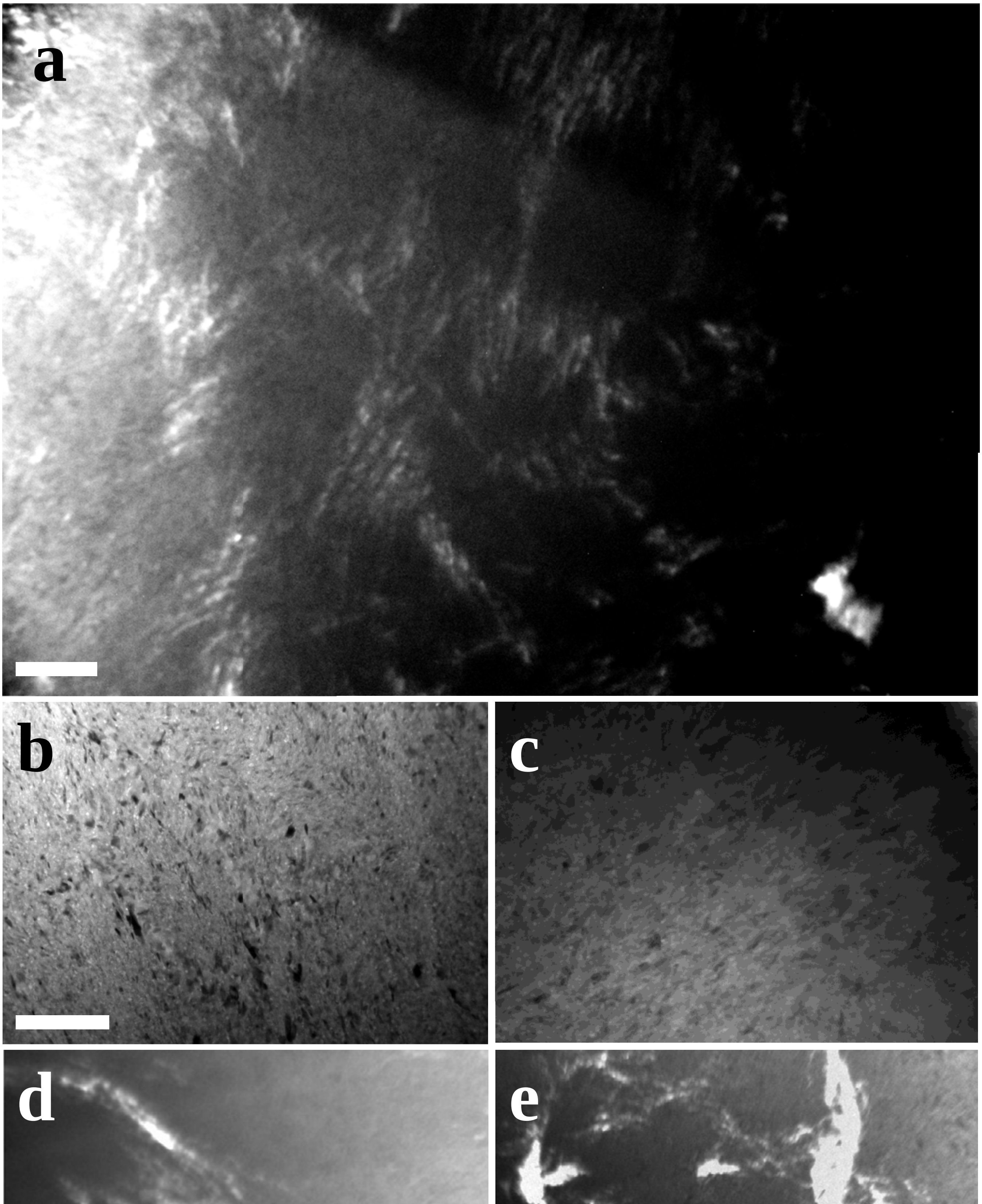

Supplementary Figure 10. TEM micrographs depicting **a,** fibre fragments found in specimen NHMUK R4243; **b, c**, area of fossilized sample with no fibres, found in NHMUK R4249 (**b**) and NHMUK R4864 (**c**). **d, e**, areas with fibres from sample NHMUK R4249. Scale bar = 500 nm.

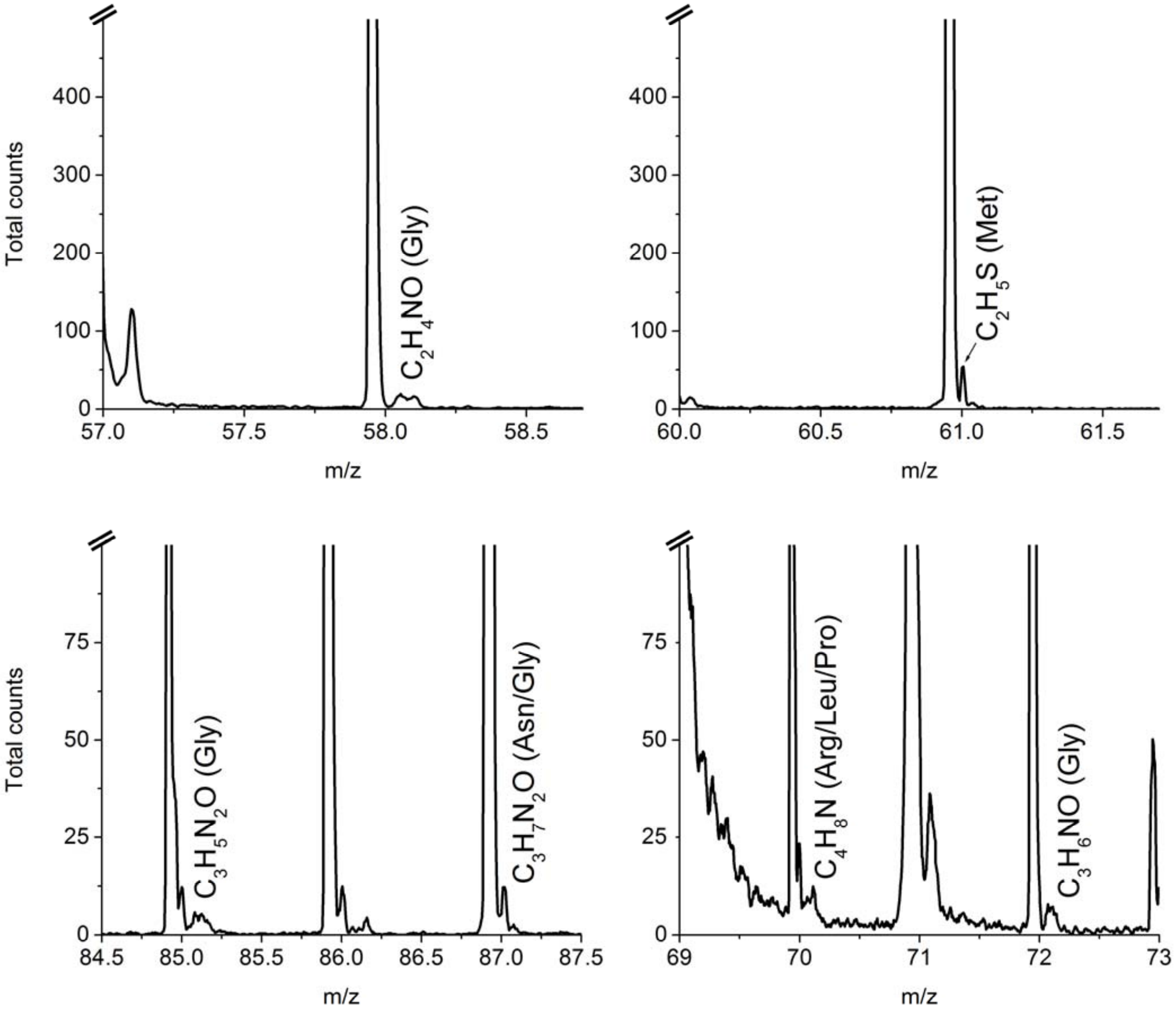

Supplementary Figure 11. Mass spectra detail obtained using an IONTOF TOF.SIMS[5]-Qtac[100] LEIS from NHMUK R4493 containing fibres extracted with FIB. Mass peaks correspond with amino acid fragments, as labelled. For assignment of peaks, see Supplementary Table 2.

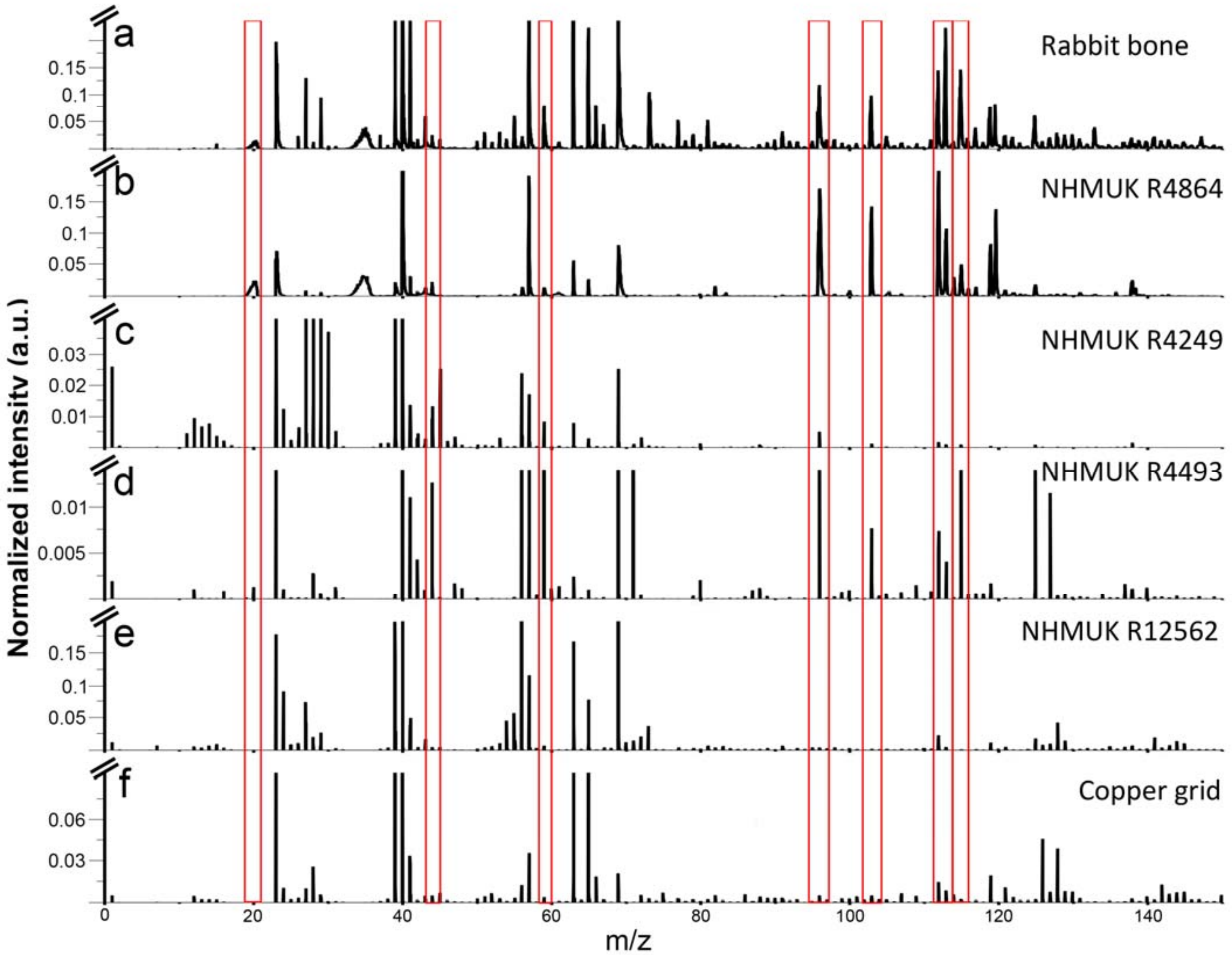

Supplementary Figure 12. Mass spectra detail obtained using an IONTOF TOF.SIMS^{5}-Qtac^{100} LEIS from **a,** rabbit bone, **b,** NHMUK R4864 (presenting calcified fibres imaged by SEM), a hadrosaurid tibia, **c,** NHMUK R4249 (presenting calcified fibres imaged by SEM and TEM and 67 nm banding imaged by TEM), an ungual phalanx of a hadrosaurid, **d,** NHMUK R4493 (presenting calcified fibres imaged by SEM and TEM and 67 nm banding imaged by TEM), rib fragments from an indeterminate dinosaur, **e,** a fossil not showing any sign of calcified fibres (NHMUK R12562) and **f,** the copper grid to which samples are attached. Red boxes highlight the peaks that are present on fossilized bone but not on the cement or the copper grid.

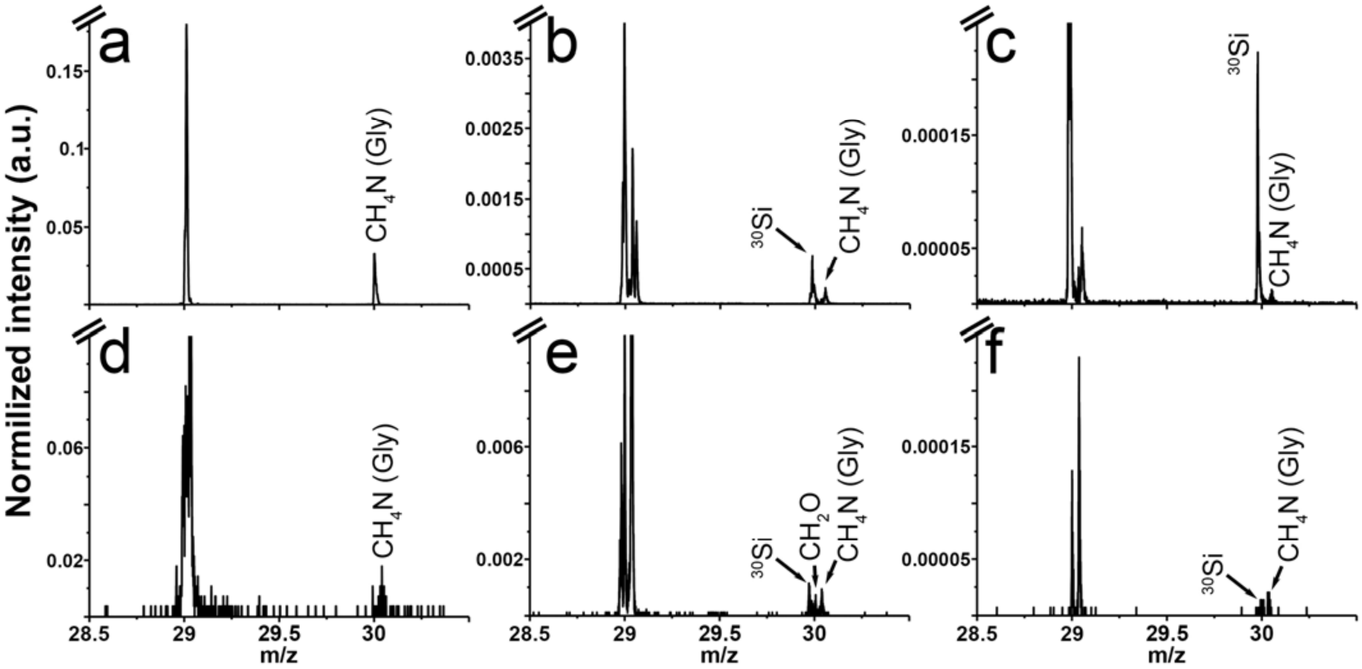

Supplementary Figure 13. Detail of mass spectra obtained using an IONTOF TOF.SIMS[5]-Qtac[100] LEIS from **a,** NHMUK R4249 (presenting calcified fibres imaged by SEM and TEM and ~67 nm banding imaged by TEM), **b,** NHMUK R4243 (presenting calcified fibres imaged by SEM and TEM and ~67 nm banding imaged by TEM), **c,** NHMUK R4493 (presenting calcified fibres imaged by SEM and TEM and 67 nm banding imaged by TEM), **d,** rabbit bone, **e,** a fossil not showing any sign of calcified fibres (NHMUK R12562) and **f,** the copper grid.

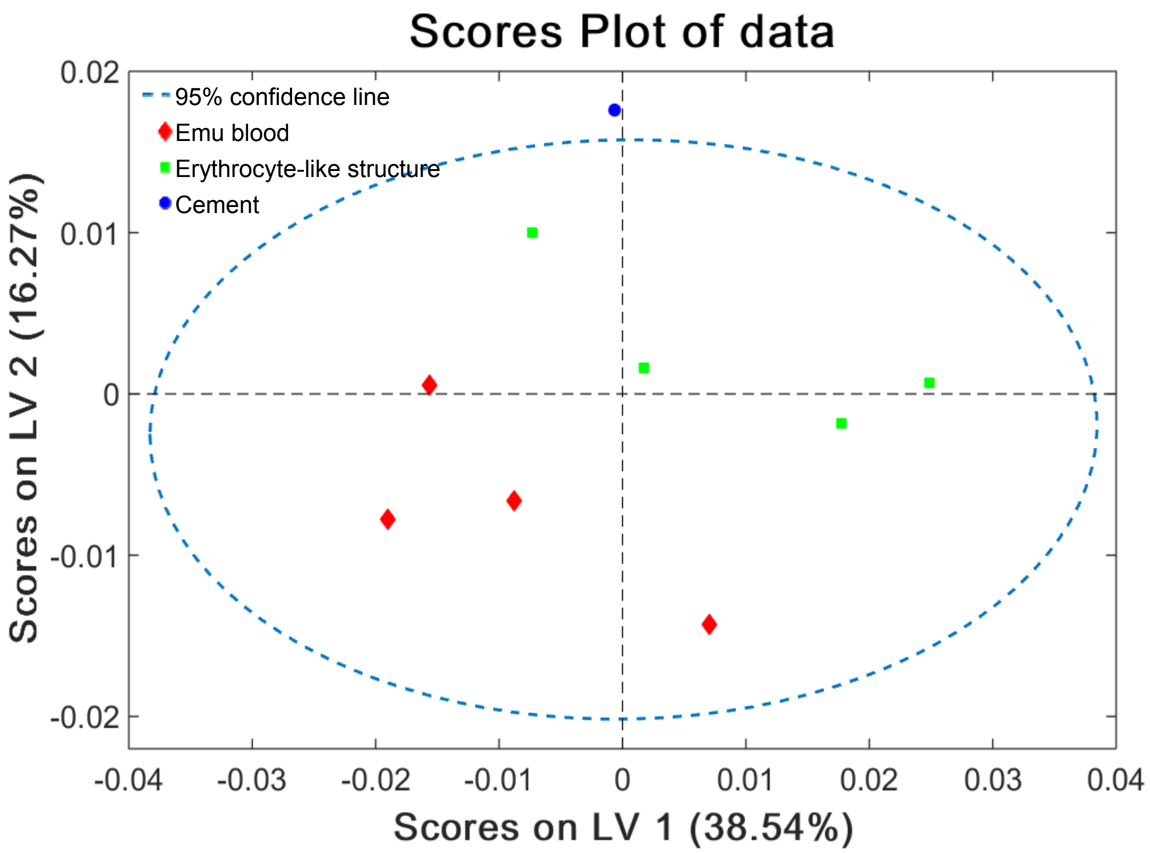

Supplementary Figure 14. Partial Least Squares – Discriminant Analysis (PLS-DA) of mass spectra from erythrocyte-like structures found on sample NHMUK R12562, emu blood samples and cement adjacent to erythrocyte-like structures. Score plot of loading vectors (LV) 1 and 2, in which each spectrum is represented by a data point. The position of each point reflects characteristic features of the spectrum. The ellipse represents the Hotellings T^2 with 95% confidence.

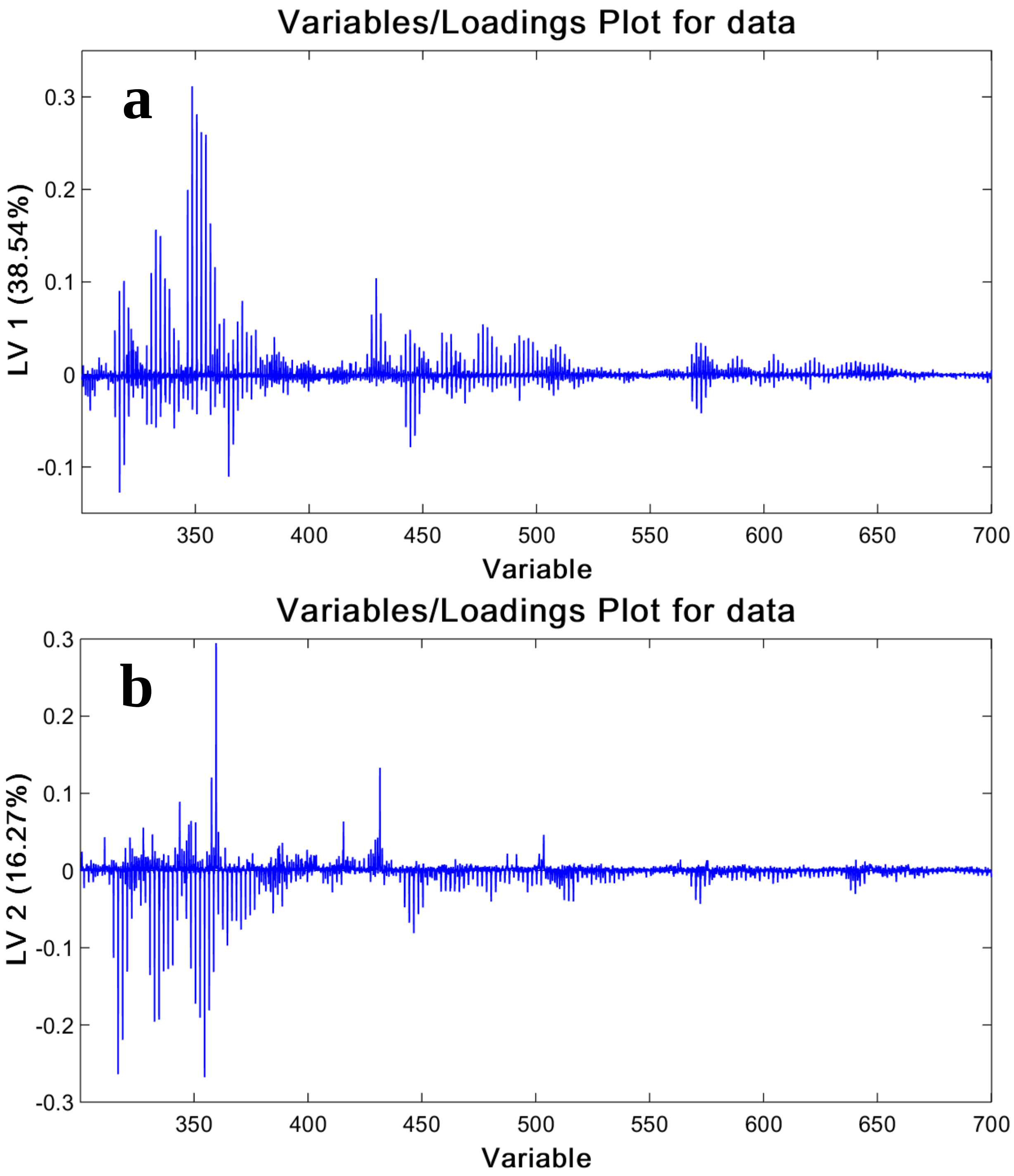

Supplementary Figure 15. Loadings plot showing origins of the differences the PLS model picked for the differentiation of the samples in supplementary Fig 14. **a,** LV1 explaining 38.54% of the variance and **b,** LV2 explaining 16.27% of the variance in the PLS model.

Supplementary Table 1: Dinosaur specimens. All specimens were collected in the early part of the 20th century and so the exact location, collection history and sedimentology of the layers in which they were found are unknown.

Specimen number	Taxonomy	Element	Locality	Collection	Structures
**NHMUK R12562**	Theropoda	Ungual claw	Dinosaur Park Formation, Alberta, Canada	Cutler Collection	Erythrocyte-like structures, amorphous carbon-rich material
**NHMUK R4948**	*Chasmosaurus*	Rib	Dinosaur Park Formation, Alberta, Canada	Cutler Collection	-
**NHMUK R4249**	Hadrosauridae	Ungual phalanx	Dinosaur Park Formation, Alberta, Canada	Cutler Collection	Fibre-like structures
**NHMUK R4243**	Hadrosauridae	Astragalus	Dinosaur Park Formation, Alberta, Canada	Cutler Collection	Carbon-rich material, Fibrous structures
**NHMUK R4864**	Hadrosauridae	Tibia	Lance Formation, Converse County, Wyoming, USA	Sternberg Collection	Fibrous structures
**NHMUK R4493**	Indeterminate	Ribs	Dinosaur Park Formation, Alberta, Canada	Cutler Collection	Collagen-like fibres
**NHMUK unregistered**	Hadrosauridae	Ischium	Unknown	Cutler or Sternberg Collection	-
**NHMUK R4484**	Ceratopsidae	Phalanx	Dinosaur Park Formation, Alberta, Canada	Cutler Collection	Erythrocyte-like structures

Supplementary Table 2: Mass spectra peak list and attribution obtained by ToF-SIMS analysis on the sections obtained from sample NHMUK R4493.

**Peak list**	**Attribution**
23	Na (sodium)
30.0	Glycine fragment - CH_4N
40.0	Ca (calcium)
43.2	Arginine fragment – CH_3N_2
44.0	Ca; Alanine fragment - C_2H_6N
56.0	Fe (iron)
56.9	Fe; CaOH; Lysine fragment – C_3H_7N
58.0	Glycine fragment - C_2H_4NO
58.9	CaF
60.9	Methionine fragment – C_2H_5S
68.3	Proline fragment - C_4H_6N
68.9	Ga (gallium)
70.2	Proline fragment - C_4H_8N
70.8	Ga (gallium)
72.0	Glycine fragment - C_3H_6NO
85.0	Glycine fragment - $C_3H_5N_2O$
87.1	Glycine fragment - $C_3H_7N_2O$
